# Congenital Defects

**Published:** 1913-11

**Authors:** 


Congenital Defects. J. H. Wood, *Med. World,* August, 1913, reports a case of anal atresia with discharge of fæces through vagina. The girl, observed since her birth in 1886, has never menstruated, but has regular molimina. He also reports a case of tough hymen. After resecting it, he could find neither uterus nor ovaries. The girl had never shown any indication of menstruating. She has since married and is apparently functionally normal, except for sterility.

